# Stressed target cancer cells drive nongenetic reprogramming of CAR T cells and tumor microenvironment, overcoming multiple obstacles of CAR T therapy for solid tumors

**DOI:** 10.21203/rs.3.rs-2595410/v1

**Published:** 2023-02-21

**Authors:** Yufeng Wang, David L. Drum, Ruochuan Sun, Yida Zhang, Ling Yu, Lin Jia, Steven J Isakoff, Allison M Kehlmann, Ali Emre Dal, Gianpietro Dotti, Hui Zheng, Cristina R Ferrone, Alphonse G Taghian, Albert B DeLeo, Hanyu Zhang, Youssef Jounaidi, Song Fan, Peigen Huang, Cheng Wang, Jibing Yang, Genevieve M Boland, Ruslan I Sadreyev, LaiPing Wong, Soldano Ferrone, Xinhui Wang

**Keywords:** stressing tumor cells, reprogramming CAR T cells, reprogramming TME, disulfiram/copper, irradiation, solid tumors

## Abstract

The poor efficacy of chimeric antigen receptor T-cell therapy (CAR T) for solid tumor is due to insufficient CAR T cell tumor infiltration, *in vivo* expansion, persistence, and effector function, as well as exhaustion, intrinsic target antigen heterogeneity or antigen loss of target cancer cells, and immunosuppressive tumor microenvironment (TME). Here we describe a broadly applicable nongenetic approach that simultaneously addresses the multiple challenges of CAR T as a therapy for solid tumors. The approach massively reprograms CAR T cells by exposing them to stressed target cancer cells which have been exposed to the cell stress inducer disulfiram (DSF) and copper (Cu)(DSF/Cu) plus ionizing irradiation (IR). The reprogrammed CAR T cells acquired early memory-like characteristics, potent cytotoxicity, enhanced *in vivo* expansion, persistence, and decreased exhaustion. Tumors stressed by DSF/Cu and IR also reprogrammed and reversed immunosuppressive TME in humanized mice. The reprogrammed CAR T cells, derived from peripheral blood mononuclear cells (PBMC) of healthy or metastatic breast cancer patients, induced robust, sustained memory and curative anti-solid tumor responses in multiple xenograft mouse models, establishing proof of concept for empowering CAR T by stressing tumor as a novel therapy for solid tumor.

## Introduction

Chimeric antigen receptor T-cell therapy (CAR T) has achieved unprecedented success as a novel immunotherapy with curative potential for certain hematologic cancers^1^. In contrast, results from clinical trials of CAR T for solid tumors have been disappointing^2,3^. Many factors contribute to the poor efficacy of CAR T for solid tumors. These include insufficient infiltration, expansion, persistence, and effector function, resulting in the ultimate exhaustion of adoptively transferred CAR T cells and an immunosuppressive tumor microenvironment (TME)^4^. In addition, intrinsic target antigen heterogeneity and/or antigen loss due to selective pressure by targeted therapies^5,6^ also contribute to the resistance of solid tumors to CAR T^7,8^. *Significant* efforts have been made to genetically engineer modified CAR T cells to promote more effective treatments for solid tumors. These include, but are not limited to, altering an array of tumor-specific CAR T cells to express (i) the p40 IL23 subunit to promote proliferation and survival^9^, (ii) the dominant-negative TGF-β receptor to promote proliferation^10^, (iii) the IL-8 receptor, CXCR1 or CXCR2, to enhance migration and persistence in the TME^11^, (iv) the anti-PD-L1 antibody, PD-1 dominant negative receptor, or PD-1–knockout alteration to block PD-1/PD-L1 signaling in CAR T cells^12,13^, and (v) immunostimulatory RNA RN7SL1 to activate RIG-I/MDA5 signaling and promote expansion and effector-memory differentiation of CAR-T cells. The cumulative effects of these alterations enhance myeloid cell and dendritic cell (DC) activity, reverse immunosuppressive TME conditions, and prime endogenous T cells to reject tumor cells with CAR-directed antigen loss^14^.

The percentage of CAR T cells with central memory phenotype (T_CM_) is highly concordant with longer-term *in vivo* persistence and favorable clinical outcomes in neuroblastoma^15^. Moreover, stem-like memory T cells (T_SCM_) have been shown to play a critical role in mediating early anti-leukemic responses and long-term immune surveillance against relapse of leukemia in patients for up to 3 years^16^. Importantly, sustained remission of chronic lymphocytic leukemia (CLL) has been associated with elevated frequency of CD19 CAR T cells with memory-like characteristics. More striking, the presence of an **early memory T cell population** in the pre-manufacturing leukapheresis product predicted, with 100% accuracy, responders vs. non-responders to CD19 CAR T therapy among CLL patients^17^. These results underscore an essential requirement for successful CAR T, namely, **a high frequency of early memory CAR T cells**. However, genetically engineering one or even several genes produces a limited effect, and it is difficult to engineer a genetic solution that simultaneously addresses all or most of the existing barriers to treating solid tumors. Moreover, even though the efficacy of these genetically modified CAR T cells is improved compared to the parental CAR T cells, they still yield low curative outcomes in preclinical models as well as in clinical trials for solid tumors^2^. Thus, we sought to develop a more broadly applicable non-genetic approach that could overcome many if not all the obstacles that prevent CAR T from achieving long-lasting complete solid tumor rejection.

Disulfiram (DSF) is an irreversible pan-aldehyde dehydrogenase (ALDH) inhibitor approved by the FDA in 1951 for treating alcoholism^18^. DSF is also a chelator and primarily complexes with Cu^2+^ (DSF/Cu)^19^. It is well established that DSF/Cu targets the p97 segregase adaptor nuclear protein localization protein 4 (NPL4), which is essential for protein turnover and involved in multiple regulatory and stress-response signaling pathways^20^. By blocking NPL4/p97, DSF/Cu activates endoplasmic reticulum (ER) stress via upregulating the inositol-requiring enzyme 1 alpha (IRE1α)–X-box-binding protein 1 (XBP1) axis, leading to autophagic apoptosis^21^. Recently, we found that by combining ionizing radiation (IR) with DSF/Cu, we induced more robust immunogenic cell death (ICD) of differentiated cancer cells and cancer stem cells than could be achieved with either method alone^22^. The molecular characteristics of ICD include the release or cell-surface expression of highly immune-stimulatory damage-associated molecular pattern molecules (DAMPs). These molecules stimulate antigen-presenting cells (APCs), which boost CAR T expansion and activity^23^ and activate dendritic cells (DCs), leading to subsequent development and activation of endogenous effector T cells and memory T cells^22,24^ (unpublished data). In addition, DAMPs function as ligands for pattern recognition receptors (PRRs) and PRR agonists, which promote expansion and effector-memory differentiation of CAR-T cells^14^. Therefore, we hypothesized that we could use DSF/Cu and IR–stressed target cancer cells to reprogram the CAR T cells into **early-memory T cells** with more robust expansion, activation, persistence, and effector function, while simultaneously converting the immunosuppressive TME to an immunostimulatory TME by inducing the “hot death” (ICD) of cancer cells and release of pro-inflammatory cytokines and chemokines.

This study investigated whether target cancer cells stressed by DSF/Cu and IR (DSF/Cu+IR) *in vitro* and *in vivo* could reprogram not only healthy donor or metastatic breast cancer patient donor–derived CAR T cells, but also the TME, thereby improving the efficacy of these effectors against solid tumors. This “one-punch” massive impact approach, which simultaneously addresses the multiple barriers to CAR T cell therapy, was assessed *in vitro* and *in vivo* by monitoring reprogrammed and non-reprogrammed CAR T cells for evidence of early memory T-phenotype switch, expansion, infiltration, effector function, and persistence; and by analyzing reprogrammed TME to identify the number of infiltrated immune cells, immune cell subtypes, cytokine/chemokine types, and levels. More importantly, CAR T cells targeting different antigens and a panel of stressed solid tumors were evaluated for curative therapeutic potential using various established tumor cell lines and patient-derived xenograft (PDX)–derived solid tumor mouse models.

## Results

### DSF/Cu and IR induces cellular stress responses in target cancer cells

**Figure 1:**
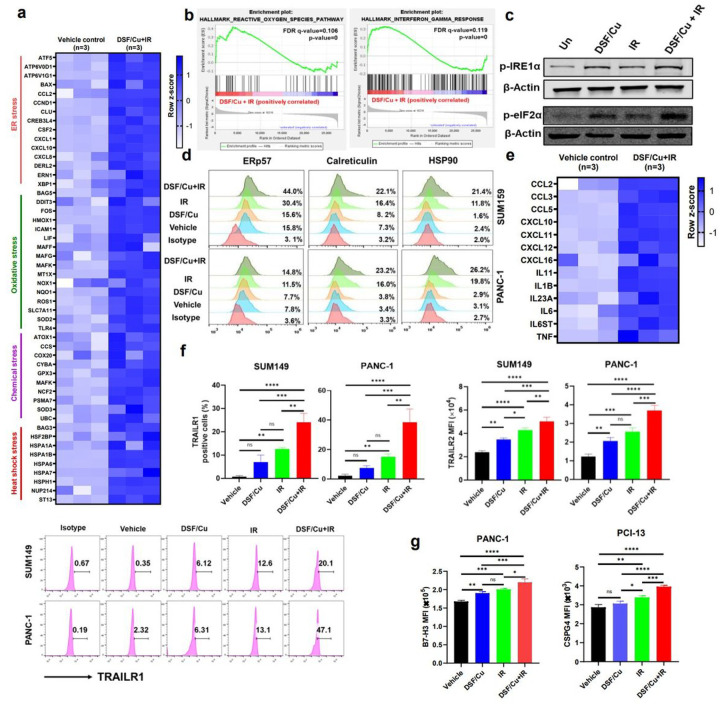
DSF/Cu+IR induces cellular stress responses in target cancer cells *in vitro*. **a,** The heatmap shows ER stress, oxidative stress, chemical stress, and heat shock stress-related gene expression between vehicle-treated SUM159 tumor cells vs. DSF/Cu+IR–treated SUM159 tumor cells (n=3). **b**,Representative GSEA enrichment plot illustrates the “INTERFERON_GAMMA_RESPONSE”, “REACTIVE_OXYGEN_SPECIES” and “INTERFERON_ALPHA_RESPONSE” (n=3). **c**, Western blot analysis of ER stress-related gene-encoded proteins p-IRE1α and p-eIF2α after indicated treatments (n=3). **d**, The expression level of stress-related markers ERp57, calreticulin, and HSP90 were measured using flow cytometry after indicated treatments (n=3). **e**, Bulk cell RNA-seq shows the upregulation of pro-inflammatory chemokine and cytokine genes in DSF/Cu+IR–stressed SUM159 cells (n=3). **f**, The percentage of TRAILR1 positive cells (SUM149 and PANC-1) after indicated treatments (n=3). **g**, The mean fluorescence intensity (MFI) values of B7-H3/CSPG4 expression on PANC-1/PCI-13 cells 24h post indicated treatments (n=3). Data are shown as the individual value and the mean ± SD. ns represents no Significant difference. * p<0.05, **p<0.01 ***p<0.001, ****p<0.0001.

Drawing on our previous work demonstrating that DSF/Cu+IR induces ICD by releasing membrane-bound and soluble factors that enhance immune cell functions^3,25^, we investigated a broad spectrum of stresses that can be induced by DSF/Cu+IR treatment of cancer cells. RNA-sequencing (RNA-seq) analysis revealed that genes related to ER, oxidative, chemical, and heat shock stress were upregulated in DSF/Cu+IR–treated vs. untreated target human breast cancer cells SUM159 (Fig1a). In addition, gene set enrichment analysis (GSEA) showed that genes of reactive oxygen species (ROS) and interferon-gamma (IFN-γ) response pathways were enriched in DSF/Cu+IR-treated target cells (Fig1b, **Extended Fig1**). Specifically, DSF/Cu+IR–induced ER stress in target cells was detected by increased p-eIF2a and p-IRE1α Fig1c) and by membrane translocation of ERp57, calreticulin (CRT), and HSP90^3,21,26^ (Fig1d). As a result, DSF/Cu+IR upregulated an array of pro-inflammatory chemokine and cytokine genes (Fig1e), increased cell membrane expression of tumor necrosis factor (TNF)–related apoptosis-inducing ligand receptor 1 (TRAILR1) and TRAILR2^27,28^ (Fig1f), and increased the CAR T cell targeted antigens, chondroitin sulfate proteoglycan 4 (CSPG4) and B7-H3 (Fig1g), all of which contributed to rendering the targeted cells more susceptible to CAR T cell-mediated killing^29–31^.

### DSF/Cu+IR-stressed cancer cells drive the phenotypic switch of CAR T cells with early memory-like characteristics

**Figure 2:**
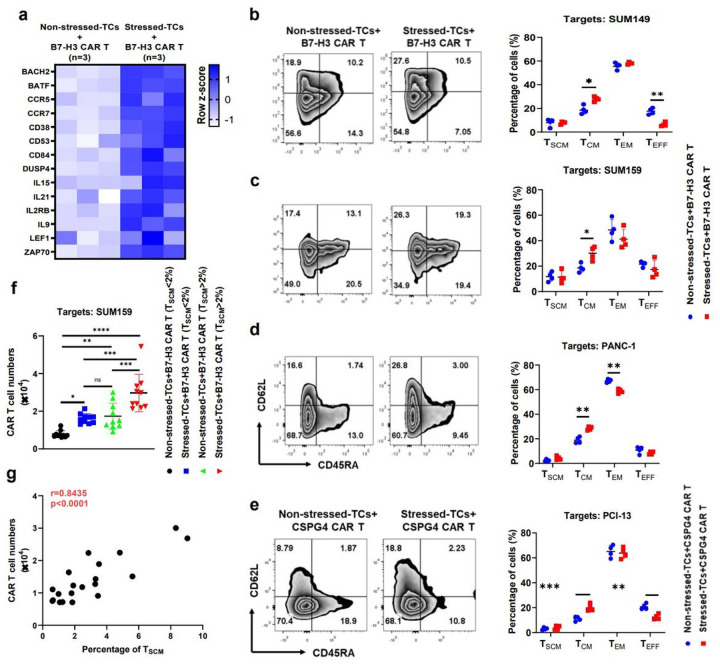
DSF/Cu+IR–stressed cancer cells drive phenotypic switch in CAR T cells with early memory-like characteristics. **a,** The heatmap shows differences in memory T cell-associated genes between B7-H3 CAR T cells co-cultured with non-stressed SUM159 tumor cells vs. DSF/Cu+IR-stressed cells (n=3). **b, c, d, e**, Phenotypic analysis of CAR T cells with markers CD45RA and CD62L after *in vitro* reprogramming by 72h co-cultured with target cells: SUM149 (**b**), SUM159 (**c**), PANC-1 (**d**) or PCI-13 (**e**). The frequencies of stem cell memory (T_SCM_, CD45RA^+^CD62L^+^), central memory (T_CM_, CD45RA^−^CD62L^+^), effector memory (T_EM_, CD45RA^−^CD62L^−^), and effector (T_EFF_, CD45RA^+^CD62L^−^) T cells in different groups are shown (n=4). **f**, The number of *in vitro* expanded CAR T cells from metastatic breast cancer patient-derived PBMCs show higher preexisting percentages of T_SCM_ (n=10) vs. lower percentages of T_SCM_ (n=10) after reprogramming by 72h co-cultured with stressed vs. non-stressed target cells (*n* =10). **g**, Positive correlation between *in vitro* expansion capacity of CAR T cells and preexisting percentage of T_SCM_ cells in PBMCs (n=20) determined by Pearson r correlation (two-tailed). Data are shown as the individual value and the mean ± SD. ns represents no Significant difference. * *p*<0.05, **p<0.01, ***p<0.001, ****p<0.0001.

Initially, we used RNA-seq analysis to assess changes in expression of memory-related genes in normal donor PBMC-derived B7-H3 CAR T cells co-cultured with **DSF/Cu+IR–stressed target cells** and compared them to B7-H3 CAR T cells co-cultured with **untreated target cells** (Fig2a). Increased expression of memory-related genes, such as BACH2, BATF, CCR5, CCR7, IL15, IL2RB, IL9, LEF1, and ZAP70, was detected in CAR T cells co-cultured **with stressed target** cells. Next, owing in part to the transcriptome changes, we looked at the percentage of central memory T cells (T_CM_), defined as CD45RA^−^CD62L^+^ cells, and found the percentage to be Significantly higher in normal donor PBMC-derived B7-H3 CAR T cells following repetitive co-culture in separate experiments with 3 types of stressed cancer cells (stressed repetitive co-culture) compared with **non-stressed cancer cells** (regular repetitive co-culture) (Fig2b, c, d). **A similar result was obtained** with normal donor PBMC-derived CSPG4 CAR T cells following repetitive co-culture with stressed vs. non-stressed tumor cells (Fig2e). Next, we confirmed the ability of DSF/Cu+IR–stressed target cancer cells to drive the phenotypic switch of CAR T cells to an early memory phenotype by examining B7-H3 CAR T cells derived from 20 breast cancer patients (**Extended-Table1**), which had been co-cultured with the stressed tumor cells. The percentage of preexisting stem-like memory T cells (T_SCM_), defined as CD45RA^+^CD62L^+^ cells, ranged from 0.61% to9.04% in PBMCs from these 20 patients. To facilitate this analysis, we categorized the findings into two groups, i.e., low T_SCM_ in PBMC (low T_SCM_/PBMC<2%) and high T_SCM_ PBMC (high T_SCM_/ PBMC>2%). The rate of PBMC-derived CAR T cells with T_SCM_ phenotype was Significantly higher in both low and high T_SCM_ groups following 48h of co-culture with DSF/Cu+IR–stressed target breast cancer cells (SUM159) compared with B7-H3 CAR T cells co-cultured with untreated non-stressed SUM159 cells. Notably, the stressed target cells drove the percentage of stem-like memory T cells (T_SCM%_) in CAR T cells derived from the low T_SCM_/PBMC group to levels as high as the non-reprogrammed CAR T cells derived from the high T_SCM_/PBMC group (Fig2f). These data indicate our novel approach to generating CAR T cell preparations, with increased cells of the T_SCM%_ or T_CM_ phenotype, may convert clinical non-responders to responders and further enhance anti-tumor response in responders to CAR T therapy. Finally, in agreement with CLL patients^17^, the levels of preexisting T_SCM_ in PBMCs from the 20 breast cancer patients were highly correlated with the expansion capacity of their CAR T cells (Fig2g).

### DSF/Cu+IR stressed-target cells promote functional switch in CAR T cells with profoundly enhanced *in vitro* expansion and cytotoxicity

**Figure 3:**
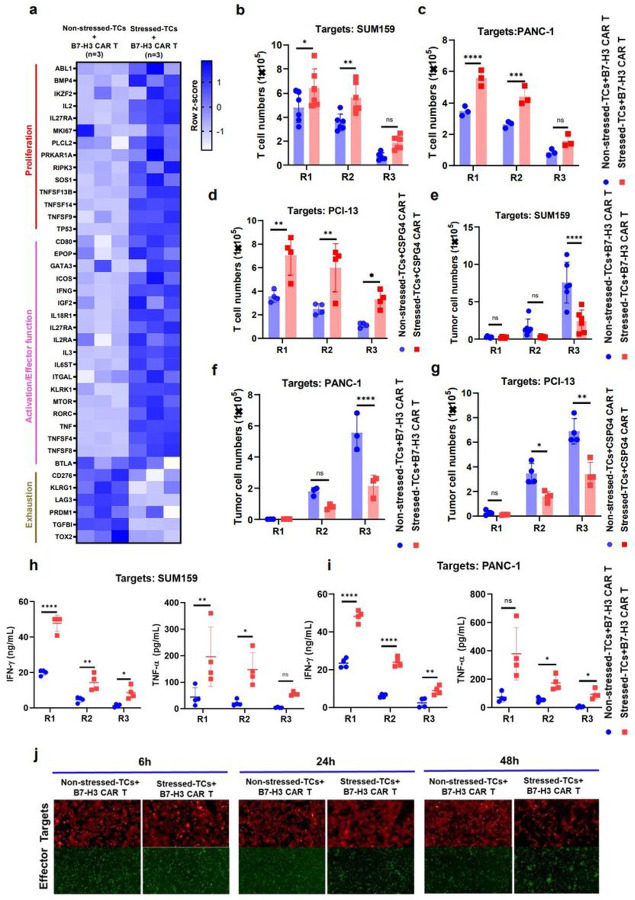
DSF/Cu+IR–stressed target cells promote functional switch in CAR T cells with profoundly enhanced *in vitro* expansion and cytotoxicity. **a,** Heatmap shows differential gene expression between B7-H3 CAR T cells reprogrammed by co-culture with non-stressed SUM159 tumor cells vs. DSF/Cu+IR–stressed cancer cells (n=3).**b, c, d, e, f, g,** The absolute number of CAR T (**b, c, d**) and tumor cells (**e, f, g**) was counted after each round of repetitive co-culture (E:T=1:2) with non-stressed vs. DSF/Cu+IR–stressed cells: SUM159 (**b, e**, n=6), PANC-1 (**d, f,** n=3) or PCI-13 (**e, g,** n=4). **h, i,** TNF-α and IFN-γ released in the supernatant collected at the end of each round of repetitive co-culture was measured by ELISA (n = 4). **j**, Representative data for CAR T cell proliferation and target cancer cell reduction after 6h, 24h, and 48h of co-culture of target cancer cells with reprogrammed vs. non-reprogrammed CAR T cells. Data are shown as the individual value and the mean ± SD. ns represents no significant difference. *p< 0.05, **p<0.01, ***p<0.001, ****p<0.0001.

Initially, we found higher levels of proliferation-related genes, such as IL2, IL27RA, MKI67, TNFSF13B, TNFSF14, TNFSF9, TP53, and T cell activation genes, such as CD80, IFNG, MTOR, ICOS, KLRK1, RORC, TNFSF4, and TNFSF8, in normal donor PBMC-derived B7-H3 CAR T cells following 24h of co-culture with **DSF/Cu+IR-stressed-target cells** (Fig3a). Next, robust and sustained *in vitro* CAR T cell expansion was detected in normal donor PBMC-derived B7-H3 CAR T cells following stressed repetitive co-culture with two different breast cancer cell lines and two different pancreatic ductal adenocarcinoma (PDAC) cell lines compared to repetitive co-culture with non-stressed tumor cells (Fig3b, c, **Extended Fig 2a, b**), as well as in normal donor PBMC-derived CSPG4 CAR T cells following repetitive co-culture with stressed vs. non-stressed triple negative breast cancer (TNBC) and *head and neck* squamous cell *carcinoma* (HNSCC) cell lines (Fig3d, **Extended Fig2c**). Then, strong and sustained cytotoxicity on a panel of different target cancer cells and increased release levels of IFN-γ and TNF-α were found in normal donor PBMC-derived B7-H3 CAR T cells or CSPG4 CAR T cells, respectively, following repetitive co-culture with stressed tumor cells (Fig3 e, f, g, h, i, **Extended Fig2d, e, f**). Moreover, following co-culture with **DSF/Cu+IR–stressed target cells** (SUM149), the profoundly enhanced proliferation and cytotoxic activity of CAR T cells was shown to be time-dependent, i.e., detected as early as 6h, and gradually became more apparent over 48h as evidenced by increased numbers of CAR T cells and fewer target cells (Fig3j). Finally, upregulation of TRAIL on CAR T cells co-cultured with **DSF/Cu+IR–stressed TNBC and a similarly stressed PDAC cell line may have contributed to enhanced target cell cytolysis (*Supplementary* Fig1)**.

### CAR T cells reprogrammed *in vivo*, using target cancer cells stressed by both intratumoral delivery of DSF/Cu and tumor localized IR, induced potent, sustained memory anti-solid tumor responses in multiple xenograft mouse models

**Figure 4:**
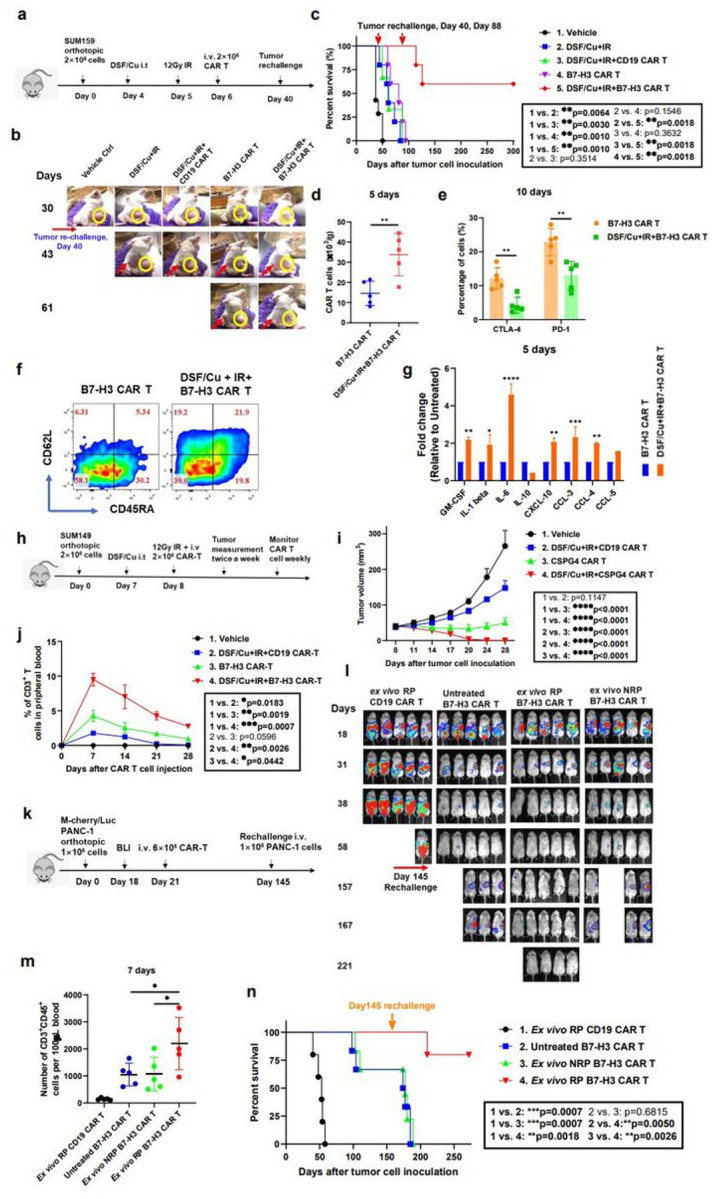
CAR T cells reprogrammed *in vivo* via target cancer cells stressed by intratumoral delivery of DSF/Cu and tumor localized IR induce potent, sustained and memory anti-solid tumor responses in multiple xenograft mouse models. **a,** Schema of the TNBC orthotopic xenograft model (SUM159) infused with CAR T cells. **b**, Representative primary and rechallenged tumors in each group at different time points. (The number of cells required to generate primary tumor and to rechallenge tumor was the same.) Yellow circle: primary tumor; Red arrow: re-challenged tumor. **c**, Kaplan-Meier survival curve of mice (n=5). Data were analyzed by log-rank test. **d**, Number of infiltrated CAR T cells in tumor tissues 5 days after CAR T cell infusion (n=5). **e**, Exhausted infiltrated CAR T cells (CD3^+^CD45^+^CTLA-4^+^ or CD3^+^ CD45^+^PD-1^+^) in tumor tissues 10 days after CAR-T cell infusion (n=5). **f**, Representative data of early memory CAR T, defined by detection of markers CD45RA and CD62L on CD3^+^CD45^+^ B7-H3 CAR T cells found in spleen 10 days after CAR T cell infusion. The percentages of stem cell memory (T_SCM_, CD45RA^+^CD62L^+^), central memory (T_CM_, CD45RA^−^CD62L^+^), effector memory (T_EM_, CD45RA^−^CD62L^−^), and effector (T_EFF_, CD45RA^+^CD62L^−^) T cells are shown. **g**, Cytokine and chemokine levels in tumor samples on day 5 after CAR T cell infusion (n=2; each sample was pooled from the tumor homogenates of 2–3 mice). **h**, Schema of the TNBC xenograft model (SUM149) infused with CAR T cells. **i**, Tumor volumes (n=5) in each group of mice bearing SUM149 cell line-derived tumors. **j**, Percentage of CAR T cells (CD3^+^) in peripheral blood collected weekly from mice bearing SUM149 cell line-derived tumors. (Blood was pooled to yield 3 samples from 5 mice/group). **k**, Schema of the orthotopic PDAC model (PANC-1) infused with CAR T cells. **l**, Orthotopic PDAC tumor burden measured by BLI (n=5). **m**, The number of CAR T cells (CD3^+^CD45^+^) in circulation at 7 days collected from mice bearing PDAC after T cell injection (n=5). **n**, Kaplan-Meier survival curve of mice bearing PDAC in each group (n=5). Data were analyzed by log-rank test. Data are shown as the individual value and the mean ± SD. ns represents no Significant difference. * *p*<0.05, **p <0.01, ***p<0.001, ****p<0.0001.

Based on promising *in vitro* data, we performed *in vivo* tests of tumor-stressing via both intratumoral (i.t.) and IR delivery of DSF/Cu to determine if we could enhance the antitumor efficacy of normal donor derived-CAR T cells in mice bearing human cancer cell line-derived xenografts (Fig4a). In the initial experiments, we treated tumors locally with DSF/Cu+IR to reprogram the systemically administered B7-H3 CAR T cells–designated RP–CAR T cells. This resulted in complete tumor regression in 100% of NSG mice bearing orthotopic TNBC SUM159 cell line-derived xenografts (Fig4b). Strikingly, when these mice were rechallenged on day 40 by inoculating SUM159 cells into the opposite lateral mammary fat pad, the tumors were gradually rejected after initially forming in all mice. In contrast, the primary and rechallenged tumors continued to grow in the B7-H3 CAR T cell (non-reprogrammed or NRP–B7-H3 CAR T cells), the DSF/Cu+IR, DSF/Cu+IR + CD19 CAR T cell, and in the vehicle-treated control groups of mice (Fig4b). Moreover, DSF/Cu+IR + RP–B7-H3 CAR T cell-treated mice exhibited long-lasting, tumor-free survival.

To further test the strength and duration of the immunologic memory response, the RP–B7-H3 CAR T cell-treated tumor-free mice were rechallenged on day 88 with SUM159 cells for the second time and 60% (3/5) of the mice rejected the challenge, maintaining long-lasting, tumor-free survival (Fig4c). To understand at least part of the reason for this durable and potent memory antitumor response, we examined the phenotypes of the CAR T cells and infiltration into tumors RP–B7-H3 CAR T cell-treated tumor xenograft mice. We found that RP–B7-H3 CAR T cell-treated mice had higher levels of splenic T_SCM_ (CD45RA^+^CD62L^+^), T (CD45RA^−^CD62L^+^CM) and tumor-infiltrated CAR T cells (CD3^+^CD45^+^), in addition to having fewer exhausted CAR T cells (CD3^+^CD45^+^CTLA-4^+^/PD-1^+^) and higher pro-inflammatory cytokines/chemokines in tumor tissues compared to mice treated with B7-H3 CAR T cells only (Fig4d, e, f, g). Next, we tested whether the same strategy could be used *in vivo* to boost the anti-solid tumor efficacy of RP–CAR T cells targeting a different antigen, i.e., CSPG4. (Fig4h) Likewise, the reprogrammed CSPG4 CAR T cell (RP–CSPG4 CAR T cell) treatment caused complete tumor regression of orthotopic TNBC SUM149 cell line-derived solid tumors in 100% of mice. In contrast, CSPG4 CAR T or DSF/Cu+IR + CD19 CAR T treatment inhibited tumor growth, but neither treatment caused complete tumor regression (Fig4i). *In vivo* expansion and persistence of CSPG4 CAR T cells (CD3^+^CD45^+^) was Significantly higher in mice treated with RP–CSPG4 CAR T cells compared to NRP–CSPG4 CAR T cells (Fig4j).

Last, we assessed whether the same strategy could boost the anti-tumor activity of B7-H3 CAR T cells in a PDAC xenograft model, namely, PDAC PANC-1 cell line-derived xenografts in mice (**Extended Fig3a**). Again, RP–B7-H3 CAR T treatment resulted in complete tumor regression in 100% of mice while NRP–B7-H3 CAR T treatment inhibited tumor growth Significantly but did not cause complete tumor regression (**Extended Fig3b**). *In vivo* expansion and persistence of B7-H3 CAR T cells at early (day 8) or late (day 92) time points after CAR T infusion of each mouse group were positively reflected in their antitumor efficacy (**Extended Fig3b, c, d**). RP–B7-H3 CAR T-treated mice had long-lasting tumor-free survival (**Extended Fig3e**). To further confirm the strength and duration of the immunologic memory response that was previously observed in the SUM159 mouse model (Fig4a, b, c, d, e, f), all tumor-free mice were rechallenged on day 135 with PDAC cells; 60% (3/5) of mice rejected the tumor rechallenge and remained long-lasting tumor-free (**Extended Fig3e**). Subsequently, we asked the question, if there is no accessible tumor for IR and /or i.t. injection of DSF/Cu, could this approach be applied *ex vivo* to enhance CAR T cell antitumor efficacy? To this end, we determined *in vivo* the anti-solid tumor activity of magnetic bead-sorted B7-H3 CAR T cells following 48h *in vitro* co-culture with PANC-1 cells pre-stressed by DSF/Cu and IR, designated *ex vivo* reprogrammed (*ex vivo* RP) CAR T cells (Fig4k). Sorted B7-H3 CAR T cells following 48h *in vitro* co-culture with untreated PANC-1 cells, designated *ex vivo* non-reprogrammed (*ex vivo* NRP) CAR T cells, and non-cocultured (untreated) B7-H3 CAR T cells served as controls.

The *ex vivo* RP–B7-H3 CAR T cells mediated complete tumor rejection in 100% of mice bearing orthotopic PDC-1 cell line-derived solid tumor xenografts, and the mice remained tumor-free for the length of the experiment. In comparison, treatment with *ex vivo* NRP and untreated B7-H3 CAR T cells resulted in Significant tumor growth inhibition or undetectable tumor by bioluminescence imaging (BLI) in only 40–60% of mice, respectively (Fig4l). *In vivo* expansion and persistence of B7-H3 CAR T cells was Significantly higher in mice treated by *ex vivo* RP B7-H3 CAR T cells compared to other groups (Fig4m). To assess whether *ex vivo* RP–B7-H3 CAR T cells could also induce an immunologic memory response equivalent to that obtained by i.t. injection of DSF/Cu and tumor localized IR, we rechallenged all mice with PANC-1 cells on day 145. A strong immunologic memory response was observed in 80% (4/5) of mice treated by *ex vivo* RP–B7-H3 CAR T cells, as measured by tumor rejection and/or remaining tumor-free, while 20% (1/5) developed tumors (Fig4n).

### Tumors stressed by DSF/Cu and IR reverse immunosuppressive TME in humanized mice

**Figure 5:**
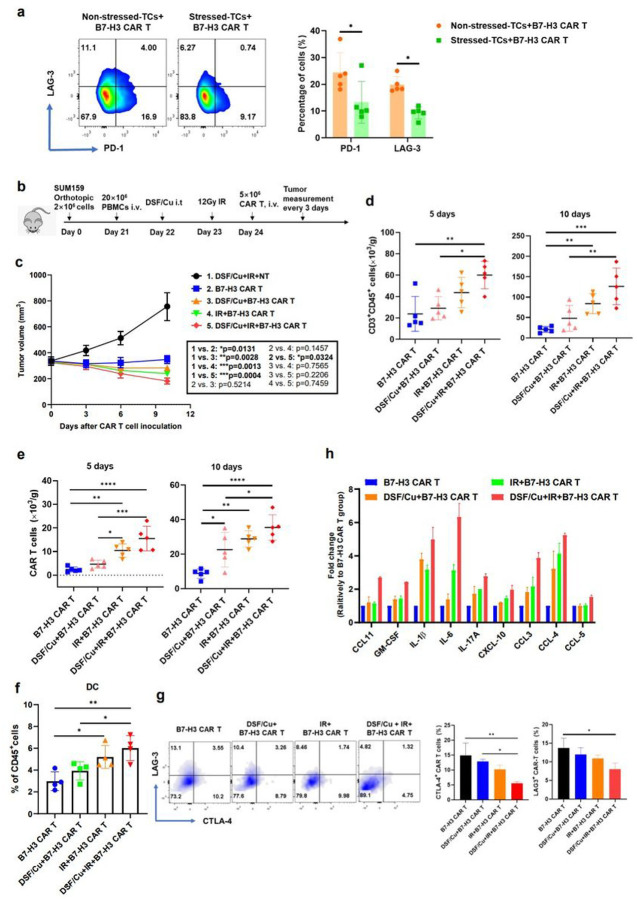
Tumors stressed by DSF/Cu and IR reverse immunosuppressive TME in humanized mice. **a**, Exhausted B7-H3 CAR T cells (CD3^+^PD-1^+^ or CD3^+^LAG-3^+^) after round 3 (R3) of repetitive co-culture with DSF/Cu+IR–stressed PANC-1 cancer cells vs. non-stressed (n=5). **b**, Schematic diagram of the humanized mouse tumor model. **c**, Tumor volumes (n=5) in the humanized mice measured every 3 days. **d**, Number of the total human CD3^+^ CD45 ^+^ cells including engrafted human PBMC and CAR T cells in tumor tissues 5 days and 10 days after CAR T cell infusion (n=5). **e**, Number of B7-H3 CAR T cells only in tumor tissues 5 days and 10 days after CAR T cell injection (n=5). **f**, The percentage of DCs (CD45^+^CD11b^+^) in tumor tissues 5 days after CAR T cell injection. **g**, Exhausted B7-H3 CAR T cells (CD3^+^CD45^+^LAG-3^+^ or CD3^+^CD45^+^CTLA-4^+^) in tumor-infiltrating CAR T cells in tumor tissues 10 days after CAR T cell infusion (n=5). **h**, Cytokine and chemokine levels in tumor tissues on day 5 after CAR T cell injection. (*Tumor homogenates were pooled resulting in 2 samples from 5 mice/group*). Data are shown as the individual value and the mean ± SD. ns represents no Significant difference. *p<0.05, **p<0.01, ***p<0.001, ****p<0.0001.

Recently, we reported that **DSF/Cu and IR–induced ICD of breast cancer cells**^22^ **can promote anti-tumor immune responses**. In this study, we found that DSF/Cu+IR upregulated an array of pro-inflammatory chemokine and cytokine genes (Fig1e), increased tumor infiltration of CAR T cells (Fig4d), and decreased levels of exhausted CAR T cells *in vitro* and *in vivo* (Fig4e, Fig5a, **Extended Fig4d, e, f, g, h**). To gain knowledge about the effect of **our approach in immunocompetent mice, we investigated DSF/Cu+IR–stressed tumor-induced TME changes in humanized mice bearing TNBC SUM159 cell-derived xenografts (Fig5b). When the average tumor reached approximately 350 mm^3^ in dimension, the** DSF/Cu+IR+B7-H3 CAR T treatment **was initiated. Owing to the rapid response to this therapy, the tumors** shrank **quickly in** mice (Fig5c). **To ensure a sufficient volume of tumor tissues would be available for subsequent analyses, specimens had to be collected on days 5 (n=5 mice/group) and 10 (n=5 mice/group) after CAR T cell infusion. At both time points, we found that** DSF/Cu+IR+B7-H3 CAR T cell-treated mice had higher levels of infiltrated T cells (CD3^+^CD45^+^)(Fig5d), CAR T cells (Fig5e), and DCs (CD11b+) (Fig5f), as well as lower levels of exhausted CAR T cells (LAG-3^+^CTLA-4^+^) in the TME (Fig5g) compared to those found in mice treated with either IR+ B7-H3 CAR T or DSF/Cu+B7-H3 CAR T or B7-H3 CAR T. In agreement with what was found in the TME of NSG mice (Fig4g), a distinctive signature of upregulated pro-inflammatory cytokines and chemokines, including IL-1β, IL-6, IL-17A, GM-CSF, CCL11, CXCL-10, CCL3, CCL4 and CCL5, was detected at high magnitude protein levels in the TME of mice treated by DSF/Cu+IR+B7-H3 CAR T cells. In comparison, levels of the above distinctive signature of cytokines/chemokines were also upregulated but to a lesser extent in TME of mice treated with IR+B7-H3 CAR T cells or DSF/Cu+B7-H3 CAR T cells (Fig5h). These pro-inflammatory cytokines and chemokines may partially account for the reprogramming initiated by DSF/Cu+IR, enabling the conversion of an immunosuppressive TME to an immune supportive TME^32,33^.

### Activation of the JAK/STAT signaling axis can be attributed to stressed cancer cell-induced phenotypic and functional switches of CAR T cells

Next, we sought to identify the main responder when CAR T cells encounter DSF/Cu+IR–stressed cancer cells. As indicated earlier, DSF/Cu+IR induces cell stress responses leading to upregulation of an array of pro-inflammatory chemokine and cytokine genes in target cancer cells (Fig1e). Of these, the following major changes attracted our attention: (i) genes leading to the activation of JAK/STAT signaling pathways (**Extended Fig5a, b**), (ii) time-dependent increases of IL-6 release, most noticeably detected in DSF/Cu+IR–stressed SUM159 cells (**Extended Fig5c**), as well as the predominant increase of IL-6 compared to other cytokines/chemokines in TME of NSG or NSG-humanized mice treated with DSF/Cu+IR (Fig4g +Fig5h), and (iii) the profound increase of IFN-γ released from CAR T cells following stressed repetitive co-culture with 2 types of target cells (Fig3h, i). It is well-established that IL-6 and IFN-γ are potent activators of the JAK/STAT pathway, which plays a pivotal role in cytokine receptor signaling and governance of T cell phenotype, proliferation, survival, and function^34,35^. These data prompted proof of principle experiments to determine if the stressed cancer cell-induced CAR T cell phenotype and functional switches are truly dependent on the IL-6-JAK/STAT signaling axis. When CAR T cells were co-cultured with DSF/Cu+IR–stressed SUM159 cells, they showed a higher level of activation of the JAK/STAT axis than when co-cultured with untreated SUM159 cells. This was indicated by upregulation in the CAR T cells of phosphorylated (p) JAK1, p-JAK2, p-STAT3, p-STAT5 and its target anti-apoptotic protein BCL-2 (**Extended Fig5d**). We then demonstrated that the JAK inhibitors (JAKi), ruxolitinib and/or momelotinib, decreased T_CM%_ in B7-H3 CAR T cells in a dose-dependent manner (**Extended Fig5e**). More important, the JAK inhibitors completely abolished, in a dose-dependent fashion, the effects of increasing T_CM%_ and expansion of B7-H3 CAR T cells by DSF/Cu+IR–stressed cancer cells (**Extended Fig5e**).

However, JAKi decreased the target cell killing capacity of B7-H3 CAR T cells, regardless of co-culture with non-stressed or stressed tumor cells (**Extended Fig5f**).

### Robust and long sustained therapeutic responses against solid tumors by RP-B7-H3 CAR T derived from PBMC of patients with metastatic breast cancer

**Figure 6:**
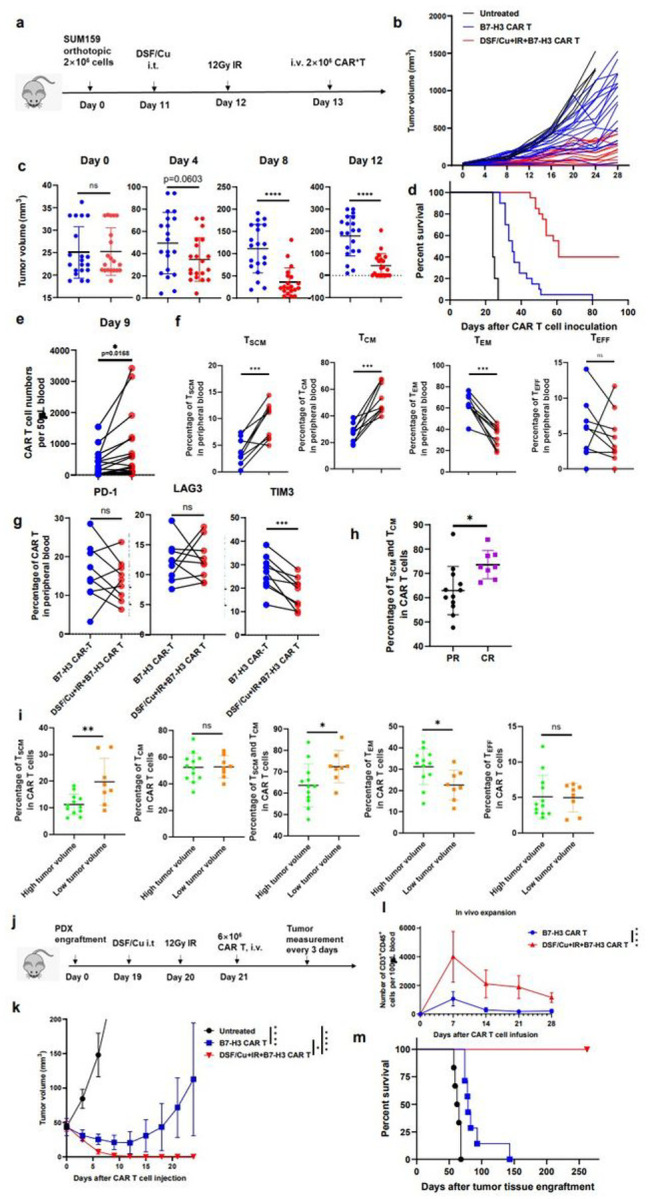
Robust and long sustained therapeutic responses against solid tumors by RP B7-H3 CAR T derived from PBMCs of patients with metastatic breast cancer. **a**, Schema of the TNBC orthotopic xenograft model (SUM159) infused with CAR T cells. **b**, Tumor volumes (n=45) of each mouse group measured every 4 days. **c**, Comparison of tumor volumes in mice treated with the same patient’s PBMC-derived CAR T vs DSF/Cu+CAR T on days 0, 4, 8 and 12 post CAR T cell inoculation. **d**, Kaplan-Meier survival curve of mice of each group (n=5 untreated, 20=n CAR T, n=20 DSF/Cu+IR+CAR T). Data were analyzed by log-rank test. **e**, Frequency of B7-H3 CAR T cells in peripheral blood 9 days post CAR T cell injection (n=40). Data were analyzed by two-tail paired t test. **f**, Early memory B7-H3 CAR T, defined by detection of markers CD45RA and CD62L on CD3^+^ in the peripheral blood collected from treated mice10 days after the CAR T cell infusion. The percentages of stem cell memory (T_SCM_, CD45RA^+^CD62L^+^), central memory (T_CM_, CD45RA^−^CD62L^+^), effector memory (T_EM_, CD45RA^−^CD62L^−^) and effector (T_EFF_, CD45RA^+^CD62L^−^) CAR T cells are shown. Data were analyzed at least 100 cells/sample by two-tail paired t test (n=18) **g**, Frequency of exhausted B7-H3 CAR T cells (CD3^+^PD-1+, CD3^+^LAG3^+^ or CD3^+^TIM3^+^ in peripheral blood collected from treated mice 9 days post CAR T cell injection(n=18). Data were analyzed by two-tail paired t test. **h**, Comparison of percentages of T_SCM_ and _TCM_ of B7-H3 CAR T cells in circulation of tumor bearing mice with complete response (CR) vs. partial response (PR) 24 days post CAR T cell infusion. (n=20) **i**, Reverse correlation of percentages of early /effector memory NRPB7-H3 CAR T cells with tumor burden (n=12 in high tumor volume group and n=8 in low tumor volume group) **j**, Schema of the TNBC PDX mouse model. **k**, Tumor volumes (n=6 Vehicle Group, n=7 for the other two groups) of each group. l, Frequency of B7-H3 CAR T cells (CD3^+^CD45^+^) in the blood of treated mice collected weekly **m**, Kaplan-Meier survival curve of mice in each group(n ≥ 6 mice/group). Data were analyzed by log-rank test. Data are shown as individual value and the mean ± SD. ns represents no Significant difference. * p<0.05, **p<0.01, *** p < 0.001, ****p <0.0001.

To address the clinical relevance of our approach to enhance the ability of CAR T to eradicate solid tumors, the therapeutic efficacy of RP–CAR T cells derived from metastatic breast cancer patients with 1.01–9.51% T_SCM_ in PBMC (**Extended Table 2**) was evaluated. The RP–B7-H3 CAR T cells and NRP–B7-H3 CAR T cells derived from each patient (n=20) were compared for therapeutic responses in the orthotopic TNBC SUM159 mouse model (Fig6a). RP–B7-H3 CAR T cells showed more robust therapeutic responses than NRP–B7-H3 CAR T cells derived from the same patients (Fig6b). Twelve days after CAR T cell infusion, complete tumor rejection was observed in mice treated with RP–B7-H3 CAR T from patients with metastatic breast cancer; 16 days after CAR T cell infusion, complete tumor rejection was found in 40% (8/20) of mice treated by RP–B7-H3 CAR T, while 5% (1/20) of the mice treated with NRP–B7-H3 CAR T cells derived from the same set of patients (Fig6c) exhibited complete tumor rejection. In summary, the 8 mice treated with RP–B7-H3 CAR T with complete tumor rejection remained tumor-free and healthy, while the other 8 mice treated with NRP–B7-H3 CAR T, derived from the same PBMCs, died (Fig 6d).

It appears that the superior anti-tumor efficacy of RP–B7-H3 CAR T cells correlates with (i) increased CAR T cell expansion *in vivo* (Fig6e); (ii) more CAR T_SCM_ and CAR T_CM_ cells, together with fewer CAR T_EM_ and CAR TEFF (Fig6f), and (iii) fewer TIM3^+^ exhausted CAR T cells (Fig6g). Importantly, significantly more CAR T_SCM_ cells and CAR T_CM_ cells were detected in peripheral blood of complete responder (CR) mice with complete tumor rejection than in partial responder (PR) mice with reduced tumor sizes in RP–CAR T cell-treated mice (Fig 6h). Consistent with this finding, in NRP–B7-H3 CAR T cell-treated mice, significantly more CAR T_SCM_ cells and CAR T_CM_ cells were detected in peripheral blood of mice with low tumor burden than in mice with high tumor burden (Fig6i). Overall, the percentage of T_SCM_ in PBMCs and in CAR T cells favorably correlated with the *in vivo* anti-tumor response of these PBMC-derived CAR T cells (**Extended Fig6a, b, c, d, e**). Furthermore, B7-H3 CAR T cells derived from PBMC with low T_SCM%_ (0.85%), obtained from a 75-year-old metastatic TNBC patient, were tested in a TNBC PDX mouse model (Fig6j, Supplementary Fig2a). To this end, B7-H3^+^TNBC PDX tissue pieces were orthotopically engrafted into a group of NSG mice (Supplementary Fig2b). Subsequent RP–B7-H3 CAR T cell treatment resulted in complete tumor rejection in 100% of the treated mice. In sharp contrast, treatment with NRP–B7-H3 CAR T cells showed non-significant tumor growth inhibition compared to untreated mice (Fig6k). *In vivo* expansion and persistence of B7-H3 CAR T cells was significantly higher in mice treated with RP–B7-H3 CAR T cells than with NRP–B7-H3 CAR T cells derived from this patient (Fig6i). Most important, the RP–B7-H3 CAR T cell-treated mice remained tumor-free for the length of the experiment (Fig6m).

## Discussion

To overcome the therapeutic barriers of CAR T for solid tumors, genetically modified CAR constructs have been selectively developed to increase CAR T cell expansion, persistence, function, and infiltration as well as to decrease CAR T exhaustion and transform the immunosuppressive TME from a “cold” to “hot” state^4^. Given the challenges and limitations of genetically engineered CAR T for solid tumors, we bypassed this approach, focusing instead on nongenetic methods to stimulate “or stress” the targeted cancer cells. As a result of our investigation, we found that CAR T cells and TME could be Significantly and beneficially reprogrammed by exposure to DSF/Cu+IR–stressed cancer cells. The cellular stresses induced by DSF/Cu+IR caused immunogenic cell death (ICD) or “hot death” of targeted cancer cells accompanied by the release of highly immune stimulatory DAMPs, pro-inflammatory cytokines, and chemokines, which in turn, yielded reprogrammed (RP) CAR T cells and TME^22^ (Fig1e).

The RP–CAR T cells were superior to CAR T cells in many of the key characteristics required for potent antitumor efficacy. For example, **healthy donor-derived** RP–CAR T cells compared to non-reprogramed (NRP) CAR T cells showed upregulated expression of memory T cell-related genes (Fig2a) **and higher percentages of** T_CM_ (Fig2b, c, d, e) or T_SCM_ (Fig4f), **leading to robust and sustained *in vitro* and *in vivo*** CAR T cell expansion (Fig3b, c, d, e, f, g, Fig4j, Fig6e, **Extended Fig2a, b, Extended Fig3c**) and greater *in vivo* persistence^15^. (Fig4c, Fig6m, **Extended Fig3e**) This finding is consistent with a recent clinical report that early memory T cells play a determinant role in the clinical success of CD19 CAR T cells in CLL^17^. Second, RP–CAR T cells acquired higher levels of expression of proliferation-related and T cell activation genes (Fig3a), greater cytotoxicity (Fig3e, f, g, h, i, j, **Extended Fig2d, e, f**), and increased release of IFN-γ and TNF-α (Fig3h, i). Third, the combination of healthy donor-derived CAR T cells and local treatment of tumors with DSF/Cu+IR resulted in the complete response of primary cell line or patient-derived TNBC or PDAC xenografts in 100% of treated mice (Fig4b, i, Fig6k, **Extended Fig3b**). Furthermore, as an indication of long and sustained immunological memory responses against tumors from the persistent residual CAR T cells, first-time rechallenged TNBC tumors were rejected in 100% of mice and PDAC tumors in 60% of mice (Fig4b, **Extended Fig3e**), while even second-time rechallenged TNBC tumors were rejected in 60% of mice (Fig4c). Finally, DSF/Cu+IR–induced ICD of cancer cells and the subsequent release of DAMPs, upregulated pro-inflammatory cytokines, and chemokines led to activated immune responses in the TME, in the absence of toxicity (Fig5h)^3,36^. Thus, the TME was reversed from “cold” to “hot”, as evidenced by increased tumor infiltration of CAR T cells, decreased levels of exhausted CAR T cells in NSG (Fig4d, e) In humanized mice, elevated infiltrated T cells (CD3^+^CD45^+^) (Fig5d), CAR T cells (Fig5e) and DCs (CD11b^+^) (Fig5f), and fewer exhausted CAR T cells (LAG-3^+^CTLA-4^+^) in humanized mice (Fig5g).

In this study, we investigated the impact of *in vivo* reprogramming of B7-H3 CAR T cells via stressed tumor in TNBC-bearing mice by evaluating the efficacy of RP–B7-H3 CAR T cells derived from 20 patients with metastatic breast cancer, most of whom were heavily pretreated (**Extended Table 2**). RP–B7-H3 CAR T treatment resulted in 8/20 mice exhibiting a complete response (CR), accompanied by an increased number of early CAR T memory cells and fewer CAR T effector memory and exhausted CAR T cells, and in 12/20 mice exhibiting a partial response (PR), while NRP B7-H3 CAR T cell-treated mice exhibited 0/20 CR, accompanied by fewer CAR T early memory cells and increased CAR T effector memory cells and exhaustion. These results establish the correlation between increased CAR T early memory cells in complete vs partial responder mice and concur with the finding, as defined by the transcriptional profiles, that early vs. late memory CAR T cellular products from CLL are associated with a clinical response^17^. Most notably, when we treated mice bearing PDX with RP–B7-H3 CAR T cells derived from a metastatic TNBC patient, CR was obtained in 100% of mice, accompanied by increased CAR T cell *in vivo* expansion and long-lasting tumor-free survival. At the same time, compared to untreated mice, PR was observed with Significant but diminished survival in mice treated by NRP–B7-H3 CAR T derived from the same patient. These data support further optimization of the *in vivo* reprogramming CAR T strategy to increase the ability to induce CR, spiking promise that *this type* of CAR T cell can offer the clinical benefit of CR for metastatic breast cancer patients.

Initially, our data indicated that activation of the JAK/**STAT signaling axis** was at least partially attributed to the reprogramming of **CAR T cells by stressed cancer cells** (Fig3h, i, Fig4g, Fig5h, **Extended Fig5a, b, c, d**). This observation was further confirmed by activating the JAK/STAT pathway in CAR T cells. Co-culturing CAR T cells with stressed cancer cells and JAKi, ruxolitinib and/or momelotinib, completely abolished the DSF/Cu+IR-induced effects of increasing T_CM%_ and cell expansion (**Extended Fig5e**). However, while JAKi inhibited the killing capacity of RP-CAR T cells, it also inhibited the killing capacity of NRP-CAR T cells to the same extent (**data were not shown**). Therefore, it appears that the mechanisms of reprogramming CAR T cells by stressed-cancer cells is not limited solely to activation of the JAK/STAT axis, warranting further investigation.

Importantly, DSF/Cu+IR–stressed cancer cells not only reprogrammed CAR T cells and TME, but also became more sensitive to killing by CAR T cells as the result of ICD, higher expression of TRAILR1 and TRAILR2, and upregulated target antigens (Fig1c, d, e, f). It is also worth noting that *ex vivo* reprogrammed CAR T cells presented potent antitumor activity *in vivo*, like that observed *in vivo* in tumor-bearing mice with the combination of intratumoral treatment with DSF/Cu+IR and systemic CAR T (Fig4l, m, n). The advantage of *ex vivo* RP–CAR T is that it can be used in cases where it is difficult to access the tumor for IR and/or i.t. injection of DSF/Cu. However, the overall effect of not having local delivery of DSF/Cu and IR, which puts stress on tumor cells and remodels the TME, remains unclear. A side-by-side study comparing both methods in terms of overall antitumor efficacy will be necessary to address this issue.

Notably, the data obtained from humanized mice demonstrate that DSF/Cu+IR–stressed cancer cells not only reprogram CAR T cells, but also enhance the infiltration of T and DC cells into tumors. These results imply that besides reprogramming CAR T cells, stressed cancer cells are highly likely to activate endogenous immune antitumor responses as the result of increased DAMPs and pro-inflammatory cytokines and chemokines present within the TME. Furthermore, when the reprogrammed CAR T cells target B7-H3, an immune checkpoint protein, even more pronounced anti-tumor immune responses are elicited^31^ (unpublished data).

Therefore, this approach may simultaneously empower both passive and active immunity. Passive immunity in the host patient is mediated by the adoptive transfer of CAR T- or T cell receptor (TCR)-engineered T cells or tumor-infiltrating lymphocytes (TILs). Active immunity occurs when endogenous T cells recognize a variety of tumor antigens to prevent immune escape from CAR T- or TCR-engineered T cell therapy aimed at one or a few tumor antigen target(s) which may be downregulated or lost^6^.

We have developed a nongenetic approach to reprogram and enhance the efficacy of CAR T cells and reverse the immunosuppressive TME by stimulating CAR T cells with DSF/Cu+IR–stressed target cancer cells. The reprogrammed CAR T cells acquire characteristics that promote therapeutic efficacy against solid tumors, including, but not limited to, robust cell expansion, long-term persistence, greater effector function, and decreased exhaustion. Intratumoral injection of DSF/Cu+ tumoral IR caused the release of elevated pro-inflammatory cytokines/chemokines, increased the infiltration of CAR T, T cells, and DC, and empowered the CAR T cells in the TME.

This unique “one-punch” massive impact approach overcomes most of the major obstacles that currently impede CAR T efficacy against solid tumors, yielding effectors that completely reject an array of distinct types of solid tumors, including TNBC, PDAC, and TNBC PDX, as observed in 100% of the mice tested in this study. In addition, the persistent residual RP–CAR T cells demonstrate long-term immunological memory responses as 60–100% of treated mice were protected from first-time tumor rechallenges and 60% were protected from second tumor rechallenges. Thus, stressing target cancer cells by DSF/Cu+IR is a timely approach with substantial translational potential for enhancing the efficacy of CAR T of solid tumors and inducing long-term immunological anti-tumor memory responses.

## Methods

### Cell lines and cell culture

Human triple negative breast cancer (TNBC) cell lines, SUM149 and SUM159, were acquired from the Duke Comprehensive Cancer Center Cell Culture Facility and Asterand Bioscience, Inc., respectively. The human pancreatic ductal adenocarcinoma (PDAC) cell line PANC-1 and the head and neck squamous cell carcinoma (HNSCC) cell line PCI-13 were purchased from American Type Culture Collection (ATCC). The human PDAC cell line PDAC-6 was established at MGH using ascites fluid from a patient with metastatic PDAC^37^. In some cases, the cell lines were stably transduced with m-cherry and firefly luciferase. The SUM149 and SUM159 cell lines were cultured in RPMI 1640 medium supplemented with 10% FBS. The PANC-1 and PDAC-6 cell lines were cultured in Dulbecco’s Modified Eagle’s Medium (DMEM) supplemented with 10% FBS. All cells were cultured at 37°C in 5% CO_2_ humidified atmosphere.

### Antibodies

R-Phycoerythrin AfiniPure F(ab’) Fragment Goat Anti-Mouse IgG (H+L) (cat#115-116-146), Allophycocyanin (APC) AfiniPure F(ab’) Fragment Goat Anti-Mouse IgG (H+L) (cat#115-136-146), Fluorescein (FITC) AfiniPure F(ab’) Fragment Goat Anti-Mouse IgG (H+L) (cat# 115-096-146) were obtained from Jackson ImmunoResearch. CD3-PE-Cy7 (cat#300420), CD8-APC-Cy7 (cat#344714), CD4-FITC (cat#357406), CD45RA-PE-Cy5 (cat#304110), CD45RO-APC (cat#304210), CD62L-PE (cat#304806), CD27-BV421 (cat#302824), CD45RA-FITC (cat#304148), PD1 (CD279) -APC (cat#329907), CCR7 (CD197) -BV421 (cat#353208), CD261 (DR4, TRAIL-R1)-PE (cat#307206), CD262 (DR5, TRAIL-R2)-APC (cat#307408), CD95 (Fas)-BV785 (cat#305646), CD127 (IL-7Rα)-PE (cat#351304), TNFα-PE (cat#502909), IFNγ-APC (cat#502512), Granzyme B-Pacific Blue (cat#515408), CD62L-BV711 (cat#304860), CD253 (TRAIL)-PE (cat#308206), CD45-PE (cat#368510), CD366 (Tim-3)-Pacific Blue (cat#345042), CD279 (PD-1)-PerCP/Cyanine5.5 (cat#367410), CD152 (CTLA-4)-BV605 (cat#369610), CD25-PE/Dazzle^™^ 594 (cat#302646), CD276 (B7-H3)-APC (cat#351005), CD11b-Pacific Blue (cat#301315), CD33-PerCP/Cyanine5.5 (cat#366616), CD163-BV785 (cat#333632) and Zombie Red^™^ Fixable Viability Kit (cat# 423110) were purchased from Biolegend. ATF-6 (cat#65880S), Phospho-eIF2α (Ser51) (cat#3398), Bcl-2 (cat#15071S), β-Actin (#3700), STAT5 (cat#25656S), Phospho-Stat5 (Tyr694) (cat#9356S), STAT3 (cat#9139S), JAK1 (cat#3344S), Phospho-Jak2 (Tyr1007/1008) (cat#3771), Phospho-Stat3 (Tyr705) (cat#9145), Phospho-Jak1(Tyr1034/1035) (cat#74129) and Jak2 (cat#3230) were obtained from Cell Signaling Technology. CD3-FITC (cat#555339) and CD28 (cat# 556620) were purchased from Biosciences, and CD3 (cat#130-093-387) from Miltenyi Biotec. Phospho-IRE1 alpha (Ser724) (cat# PA585647) and FITC-Labeled Human B7-H3 (4Ig) / B7-H3b Protein (cat# B7B-HF2E7-25ug) were obtained from Invitrogen and Acro Biosytems, respectively.

### Animals

The 6-to-8-week-old NSG mice were obtained from the Massachusetts General Hospital (MGH) COX7 animal facility or Jackson laboratory. The MGH Institutional Animal Care and Use Committee approved the animal studies described herein.

### *In vitro* stressing of target cells

Tumor cells were plated in 6-well culture plates at a density of 3×10^5^ cells/well (SUM149, SUM159) or 4×10^5^ cells/well (PANC-1, PDAC-6) in 2 mL RPMI 1640 medium containing 10% FBS and permitted to grow overnight. The following day, DSF/Cu (0.2 μM/1 μM) or DMSO was added and cultured for 24h, followed by 12 Gy IR. The cells were then resuspended, washed twice with PBS, and set up immediately in 6-well plates at a density of 5×10^5^ cells/well. After 6–12 h, the tumor cells were co-cultured with CAR T cells at different E:T ratios.

### IR treatment *in vitro* and *in vivo*

For the *in vitro* treatment, cells were irradiated with a single dose of IR (12 Gray (Gy)). For tumors treated *in vivo*, one 12 Gy fraction was delivered locally to each mouse over the area of the tumor, while the remaining body was shielded with a lead drape. The X-RAD 320 Biological Irradiator (Precision X-ray Inc., CT) was used for these experiments.

### Flow cytometry

For cell surface staining, the cells were harvested, washed twice with PBS, and then incubated with an appropriate amount of fluorochrome-conjugated or unconjugated antibodies for 20 min or 60 min at 4°C in 2% PBB (2% BSA in PBS buffer). Fcγ fragment-specific secondary antibodies were used accordingly if necessary. For intracellular staining, the surface-stained cells were fixed and permeabilized in Fixation/Permeabilization solution (BD Biosciences) for 20 min at 4°C followed by two washings with Perm/Wash buffer (BD Biosciences). After intracellular staining was performed, the cells were incubated for 30 min at 4°C in Perm/Wash buffer followed by two more washings with Perm/Wash buffer. To measure the number of CAR T cells that had infiltrated tumor tissues and spleens, the tumor samples were digested with collagenase IV (0.5 mg/ml; Sigma-Aldrich) and deoxyribonuclease (DNase) I (0.2 mg/ml; Sigma-Aldrich) for 30 min at 37°C. Tumor digests and spleens were filtered through 70 μm cell strainers to obtain a single-cell suspension. ACK lysing buffer (Themo Fisher) was used to lyse the red blood cells according to the manufacturer’s instructions. Viability dye was used in each sample. The cells were analyzed by flow cytometry using the LSR II cytometer (BD Biosciences) or BD Accuri C6 flow cytometer (BD Biosciences). The data were analyzed with FlowJo software.

### *In vitro* co-culture experiments

The stressed target cells were resuspended, washed with PBS twice, and set up immediately in 6-well culture plates at a density of 5×10^5^ cells/well (SUM149, SUM159, PDAC-6, PCI-13) or 7.5×10^6^ cells/well (PANC-1) in 2mL complete CAR T medium^31^. After 6–12 h, the tumor cells were co-cultured with CAR T cells at the indicated E:T ratios.

### Repetitive co-culture assay

The tumor cells (untreated, DSF/Cu+IR-stressed) were seeded in 6-well plates at a density of 5×10^5^ cells/well (SUM149, SUM159, PDAC-6, PCI-13) or 7.5×10^6^ cells/well (PANC-1) in 2 mL of complete CAR T medium and grown overnight. Then, 2.5×10^5^ CAR T cells (E: T=1:3 or 1:2) were added to the tumor cells. The CAT T cells eliminated 100% of the tumor cells within 2–5 days of co-culture (Round 1, R1). Next, the CAR T cells were collected and resuspended in 2 mL complete CAR T medium and transferred to wells containing either 5×10^5^ (SUM149, SUM159, PDAC-6, PCI-13) or 7.5×10^5^ (PANC-1) untreated tumor cells that had been seeded 12–14 h previously, and the co-culture was continued for 3 days. (Round 2, R2). The procedure was repeated once more (Round 3, R3), and the cells remaining from R3 were then collected for analysis of exhaustion-related markers (PD-1, LAG-3, Tim3).

### Phenotypic analysis of CAR T cells

To evaluate the phenotypic transformation of CAR T cells *in vitro*, cells from R1 of the repetitive co-culture assay were resuspended and washed twice with PBS. Thereafter, the cells were cultured in 2mL complete CAR T medium supplemented with 5 ng/mL IL-15 and 10 ng/mL IL-7. After 3 days, the cells were collected and stained with CD3, CD4, CD8, CD62L, and CD45RA antibodies and then analyzed using flow cytometry.

### *In vitro* cytotoxicity and expansion of CAR T cells

Cells collected at the end of the co-culture assays were stained to quantify tumor cells with B7-H3-specific mAb376.96 and CAR T cells with CD3-specific antibody. Counting beads (Thermo Fisher Scientific) were used based on the manufacturer’s instructions to obtain the absolute numbers of both residue tumor cells and CAR T cells.

### ELISA

Cell culture supernatants were quantified for secreted cytokines (TNF-α, IFN-γ) according to the manufacturer’s instructions using ELISA kit (Biolegend).

### Quantification of cytokines and chemokines

Human cytokines and chemokines found in the cell culture supernatant and tumor tissue homogenates were quantified using a customized 25-plex Luminex panel (Thermo Fisher Scientific) containing the following analytes: CCL11, GM-CSF, Granzyme B, HSP60, IFNα, IFNγ, IL-1, IL-3, IL-4, IL-6, IL7, IL8 (CXCL8), IL-9, IL-10, IL-12p70, IL-15, IL-17A, IL-18, CXCL10, M-CSF, CXCL9, CCL3, CCL4, CCL5, TNFα. Data were acquired on a MAGPIX instrument and analyzed using the ProcartaPlex analysis app.

### Preparation of cell lysate

The tumor tissue was dissected and washed briefly with chilled PBS. The samples were placed in vessels containing lysis buffer, RIPA buffer (Thermo Fisher Scientific) and 1/50 (vol./vol.) of protease inhibitor (Thermo Fisher Scientific), and then disrupted for 30s in the homogenizer. The cultured cells were detached and washed briefly with chilled PBS before adding the lysis buffer. All samples were placed on ice for 30 min and then centrifuged for 20 min to collect the supernatant without disturbing the debris. The protein concentration was quantified using a Pierce BCA Protein Assay Kit (Thermo Fisher Scientific) according to the manufacturer’s instructions.

### Western blotting

Performed as described^38^.

### RNA extraction

Total RNA was extracted using RNeasy Mini Kit (Qiagen) according to the manufacturer’s instructions.

### Bulk RNA-seq and GSEA analysis

The total RNA content of both tumor cells and CAR T cells was extracted as described above, and the library was prepared by LC Sciences company. Raw data from both tumor cells and CAR T cells was normalized in three replicates for GSEA analysis. GSEA was performed using the GSEA_4.1.0 software (Broad Institute) on genes that differentially expressed on non-stressed vs. stressed-SUM159 cells; or NRP B7-H3 CAR T cells vs. RP B7-H3 CAR T cells.

### Immunohistochemistry

Performed as described^39^.

### Generation of CAR T cells

CAR T cells were generated as described^31^.

### Isolation of reprogrammed CAR T cells

CAR T cells were co-cultured with DSF/Cu and IR-stressed tumor cells for 48 h to allow for reprogramming. The CAR T cells were subsequently isolated from the co-cultures as CD3^+^ T cell Dynabeads (catalog# 11033, Themo Fisher) via positive selection. The magnetic bead isolated CAR T cells were used in Western blotting or *ex vivo* experiments in mice.

### *In vivo* solid tumor mouse models

Five distinct xenograft models were used in this study:

- TNBC cell line-derived orthotopic mouse model: SUM149 (2×10^6^) or SUM159 (2×10^6^) tumor cells in 100 μL FBS-free RPMI medium were injected into the mammary fat pads of 6-to-8-week-old female NSG mice. When the tumors became palpable, the mice were divided into groups using a stratified randomization strategy (n = 5 mice/group) and treated as indicated.
- PDAC xenograft mouse model: PANC-1 (2×10^6^) tumor cells in 100 μL FBS-free RPMI medium were injected subcutaneously (s.c.) into the right thigh of 8-to-9-week-old male NSG mice. When the tumors became palpable, the mice were divided into groups using a stratified randomization strategy (n = 5 mice/group) and treated as indicated.
- PDAC orthotopic xenograft mouse model: mcherry/Luc-PANC-1 (2×10^6^) tumor cells were orthotopically engrafted into the pancreas of 8-to-9-week-old male NSG mice. Tumors were measured by bioluminescence imaging (BLI) and divided into groups using a stratified randomization strategy (n=5 mice/group).
- Humanized mouse bearing TNBC orthotopic tumor model: SUM159 (2×10^6^) tumor cells in 100 μL FBS-free RPMI medium were injected into the mammary fat pads of 6-to-8-week-old female NSG mice. After 21 days, the mice were divided into groups using a stratified randomization strategy (n = 10 mice/group) and humanized by i.v. injection of 2×10^7^ human PBMCs per mouse from a heathy donor, and the mice were treated with the same donor-derived CAR T as indicated.
- TNBC-PDX orthotopic mouse model (Model ID: TM00096, The Jackson Laboratory, 1^st^ passage): A 3×3×3 mm piece of *in vivo* expanded PDX was orthotopically engrafted into the mammary fat pad of 6-to-8-week-old female NSG mice. When tumors developed, the mice were divided into groups using a stratified randomization strategy (n ≥ 6 mice/group) and treated as indicated. Tumor volumes were measured using a digital caliper and calculated as described^31^.

### *In vivo* stressing of tumors by intratumoral delivery of DSF/Cu and IR

Treated tumors were intratumorally injected (i.t.) at multiple sites with 200 μL DSF/Cu (1.5 μM/1 μM) or vehicles (DMSO/PBS), followed by 12 Gy local IR on the next day.

### Systemic CAR T therapy

Freshly thawed and expanded (within 2–3 days) CAR T cells were injected i.v. into the mouse tail vein once at the indicated dose.

### Detecting phenotypes and expansion of CAR T *in vivo*

Fifty μL of peripheral blood was collected once a week from each mouse, after which red blood cells were removed using ACK lysing buffer (Themo Fisher). T cells were assessed with CD3 and CD45 antibodies or CD62L, CD45RA, PD-1, LAG3, and Tim3 antibodies and quantified using CountBright Absolute Counting Beads (Thermo Fisher) on a BD LSRII flow machine.

### Statistics

Statistical analysis was performed using GraphPad Prism v.8 software. The one-way ANOVA was used to determine the comparisons among three or more groups at a certain condition. The student’s t-test was applied to the comparison between two groups. Additional two-way ANOVA was adopted as specified. Overall survival was calculated using Kaplan-Meier methods and log-rank tests. All data are expressed as mean ± standard deviation (SD) unless specified otherwise. Results were obtained from two or three independent experiments. Differences between groups were considered Significant when p<0.05.
